# A Large-Area and Nanoscale Graphene Oxide Diaphragm-Based Extrinsic Fiber-Optic Fabry–Perot Acoustic Sensor Applied for Partial Discharge Detection in Air

**DOI:** 10.3390/nano10112312

**Published:** 2020-11-22

**Authors:** Shuchao Wang, Weigen Chen

**Affiliations:** State Key Laboratory of Transmission & Distribution Equipment and Power System Safety and New Technology (Ministry of Education), School of Electrical Engineering, Chongqing University, Chongqing 400044, China; 15340582905@163.com

**Keywords:** Fabry–Perot, single-mode fiber, graphene oxide, diaphragm, acoustic sensing, partial discharge

## Abstract

This article presents an extrinsic fiber-optic acoustic sensor applied for partial discharge (PD) detection in air. A Fabry–Perot (F-P) cavity consisting of a single-mode fiber (SMF) and a graphene oxide (GO) film, whose thickness and effective vibration diameter are approximately 500 nm and 4.377 mm, respectively, is used as this sensing core, and the manufacturing process of GO diaphragms and this sensing probe is illustrated to be simple and controllable. Performance tests indicate that this proposed sensor maintains a linear acoustic-pressure response and a flat frequency response in the range of 200 Hz to 20 kHz, while being an omnidirectional sensor and having high working stability during a ten-day test period. Additionally, PD detection results show that the minimum PD size detected by this proposed sensor in air was approximately 100 pC, which demonstrates that this proposed sensor can achieve high-sensitivity PD detection in air.

## 1. Introduction

High-sensitivity detection of partial discharges (PDs) occurring in power equipment insulation can help testing personnel find latent faults earlier, thereby allowing related solutions to be implemented earlier [1,2]. Therefore, it has important practical significance for insulation status-correct assessment of power equipment to effectively detect PDs as an important symptom and main manifestation of power equipment insulation deterioration [3,4,5], and then, it is especially important to detect PDs in air with high sensitivity because this is a basis for detecting PDs occurring in other insulating media [6,7].

Currently, fiber-optic Fabry–Perot (F-P) sensors based on the optical interference sensing principle are very popular sensors used in the detection of some key parameters in the field of power engineering [8,9,10]. In fact, fiber-optic F-P sensors can be divided into intrinsic and extrinsic sensors [11,12,13,14], and at present, the latter can be considered as the most extensive and popular fiber-optic F-P sensors owing to their simple structure, easy design and manufacturing process, and so on [15,16,17,18]. Additionally, extrinsic sensors have been mainly used in any research field needing acoustic sensing technology [19,20,21,22,23]. In fact, PDs can instantly compress surrounding media, thereby making surrounding media vibrate, which can produce acoustic signals [6,7]. Therefore, this type of sensor is very suitable for PD detection, in theory.

In this article, we used a liquid deposition method to make nanoscale graphene oxide (GO) films, and due to the basically non-conductive, excellent membrane-forming ability, good adhesion and high mechanical strength of the GO materials [24,25,26,27], an F-P cavity consisting of a single-mode fiber (SMF) and a GO diaphragm was used as the sensing core, thereby producing the proposed sensor applied for PD detection in air. In fact, the sensitivity of the sensor itself is different from that of PD detection [1,2,3,4,5,6,7,8], so the innovation of this work is mainly reflected in the excellent performance and high-sensitivity PD detection of the proposed sensor.

## 2. Materials and Methods

### 2.1. Structural Design of the Sensing Probe

**Figure 1:**
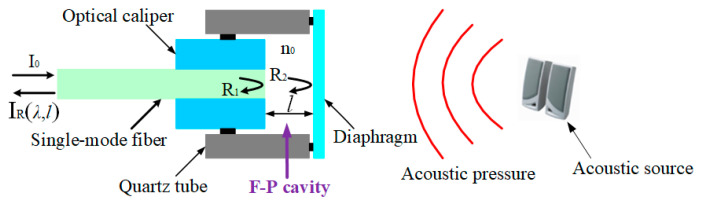
Sensing principle of the extrinsic fiber-optic Fabry–Perot (F-P) acoustic sensor.

The basic sensing principle of the extrinsic fiber-optic F-P acoustic sensor is shown in Figure 1. It can be seen from Figure 1 that one part of the incident light can be reflected at the end face of the SMF inserted into optical caliper, and the other part of the incident light can continue to propagate into the F-P cavity; then, this part of the light can be partially reflected at the inner surface of the sensing diaphragm and a portion of the reflected light can be coupled into this SMF again, thereby producing a phase shift with F-P cavity length information. Additionally, the light entering the SMF again can interfere with the reflected light from the end face of the SMF inserted into optical caliper. Therefore, when acoustic signals cause the sensing diaphragm to vibrate, the F-P cavity length can change, thereby causing the interference fringe phase to change. Then, acoustic-pressure information can be obtained by measuring the phase or intensity change of the final reflected light.

Therefore, according to references [17,18,19,20,21,22,23], the basic sensing principle of this type of sensor can be expressed by the relationship between the final reflection spectrum and the incident light wavelength, namely

(1) $$R\left(\lambda ,l\right)=\frac{I_{R}\left(\lambda ,l\right)}{I_{0}}=\frac{R_{1}{+R}_{2}-2\sqrt{R_{1}R_{2}}\cos\left(\frac{{4\pi n}_{0}l}{\lambda }\right)}{{1+R}_{1}R_{2}-2\sqrt{R_{1}R_{2}}\cos\left(\frac{{4\pi n}_{0}l}{\lambda }\right)}$$

In expression (1), λ represents the incident light wavelength; I_0_ represents the incident light intensity; I_R_(λ,*l*) represents the reflected light intensity; R_1_ represents the end face reflectance of the SMF; R_2_ represents the inner surface reflectance of the sensing diaphragm; n_0_ represents the refractive index of air in the F-P cavity; *l* represents the F-P cavity length.

According to a large number of experimental results, large-area GO films with a thickness of much less than 500 nm cannot be suspended for a long time. Therefore, we have used GO films with a thickness of approximately 500 nm as the sensing diaphragms of this proposed sensor.

In addition, according to references [20,21,22,23,24,25,26,27], the sensitivity of diaphragm can be expressed as

(2) $$S=\frac{\left|\Delta l\right|}{P}=\frac{3\left({1-\mu }^{2}\right)}{{16\mathrm{Eh}}^{3}}R^{4}$$

In expression (2), P represents acoustic-pressure size; △*l* represents the changed amount of the F-P cavity length; E represents the Young’s modulus of diaphragm; μ represents the Poisson’s ratio of diaphragm; R represents the effective vibration radius of diaphragm; h represents the diaphragm thickness.

It can be seen from expression (2) that the longer the effective vibration radius of the diaphragm, the higher the diaphragm sensitivity, and the thinner the diaphragm thickness, the higher the diaphragm sensitivity. Therefore, in this article, the effective vibration radius and thickness of the used GO-sensing diaphragm were approximately 2.2 mm and 500 nm, respectively, and they were larger and thinner, respectively, compared with other extrinsic F-P sensors [15,16,17,18,19,20,21,22,23]. Additionally, the Young’s modulus and Poisson’s ratio of GO materials are small and large, respectively [24,25,26,27]. Thus, the used GO sensing diaphragm can have very high sensitivity.

### 2.2. Production of the Sensing Probe

Here, we used a liquid deposition method to make nanoscale GO films. At first, we used the Hummer method [24,25,26,27] to prepare GO powders. Firstly, we mixed 2 g graphite powders produced by Tanfeng technology company in China with 1 g NaNO_3_ powders evenly, and then we placed them in 80 mL concentrated H_2_SO_4_ in an ice bath. The liquid was vigorously stirred while keeping the liquid temperature below 20 °C, and 8 g KMnO_4_ was slowly added to the liquid. Then, the mixed liquid was transferred to a thermostatic water bath at 35 °C and stirred for 2 h, and we added 240 mL ultra-clean water to the resulting brown paste mixture after all the above steps had been completed. In fact, the mixture can release a lot of heat at this time, so the ultra-clean water needed to be added slowly, and the mixture temperature needed to be kept below 50 °C. We added 5 mL 30% H_2_O_2_ to the mixture after the ultra-clean water was completely added, and the mixture color gradually turned bright yellow. Finally, the mixture was washed and filtered after 2 h of stirring, and then the mixture was dried in a vacuum environment to obtain GO powders.

After the GO powders were obtained, these powders needed to be peeled into flakes and dissolved in ultra-clean water by ultrasonic vibration, thereby preparing GO aqueous solution with a concentration of 780 mg/L.

**Figure 2:**
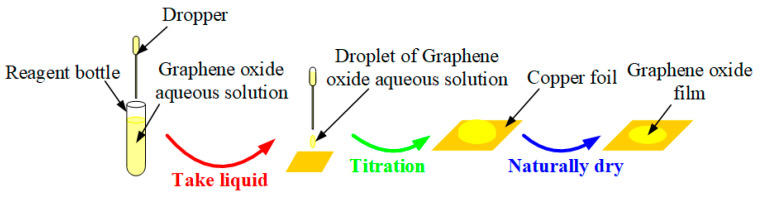
Production of graphene oxide (GO) films with copper substrates.

In addition, copper foils with a thickness of 10 μm were used as substrates during the fabrication of these sensing diaphragms. Then, we followed the steps in Figure 2 to make large-area GO films. At first, we used a 0.5-mL dropper to draw GO aqueous solution with a concentration of 780 mg/L from a reagent bottle, and then we dropped GO aqueous solution onto copper foils. Then, these copper foils were left to stand for 24 h in order to allow the water to dry naturally. Finally, we obtained GO films with copper substrates.

**Figure 3:**
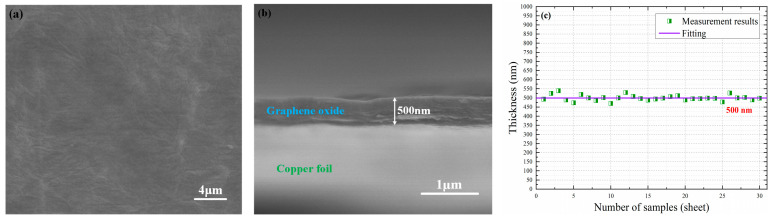
(**a**) Scanning electron microscope (SEM) image of the prepared GO film surface. (**b**) SEM image of the prepared GO film thickness. (**c**) Representative thickness values of 30 different GO films.

Figure 3a shows the prepared GO film surface situation under a scanning electron microscope (SEM, Quattro S, Thermo Fisher Scientific, Waltham, MA, USA). It can be seen from Figure 3a that the prepared GO film surface is relatively uniform and has no obvious defects, and the surface can become the inner surface of the sensing diaphragm; thus, Figure 3a indicates that the prepared GO film is very suitable as the sensing diaphragm. In addition, in order to measure thickness value of the prepared GO film, we firstly cut a border on a copper foil with the prepared GO film—a portion of the prepared GO film could be suspended at this border—and then we placed the copper foil vertically into an SEM. The measurement result is shown in Figure 3b. It can be seen from Figure 3b that the thickness value of the prepared GO film is approximately 500 nm.

Furthermore, we took 20 points on a cross-section of the prepared GO film and measured their values under SEM, and then we took the average value of these 20 thickness values as the representative thickness value of this prepared GO film; moreover, we used the method to obtain representative thickness values of 30 different GO films, and these representative thickness values are shown in Figure 3c. It can be seen from Figure 3c that the GO film thickness values prepared by GO aqueous solution with a concentration of 780 mg/L can, indeed, be considered as approximately 500 nm.

**Figure 4:**
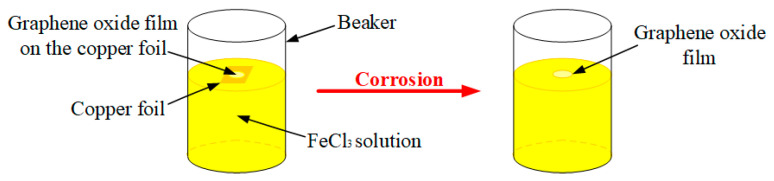
Corrosion of copper substrates.

**Figure 5:**
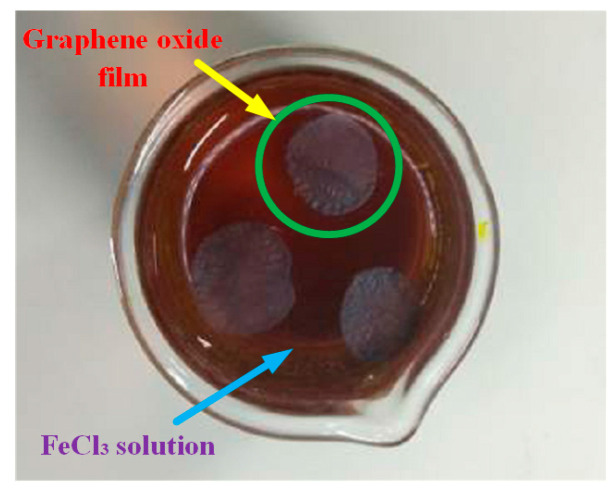
Unsupported GO films.

Then, in order to obtain unsupported GO films, copper substrates of GO films needed to be etched away, as shown in Figure 4. Firstly, we gently placed GO films with copper substrates into a beaker filled with FeCl_3_ solution and let them rest on the FeCl_3_ solution surface for 2 h to completely corrode these copper substrates. Finally, we obtained unsupported GO films, as shown in Figure 5.

**Figure 6:**
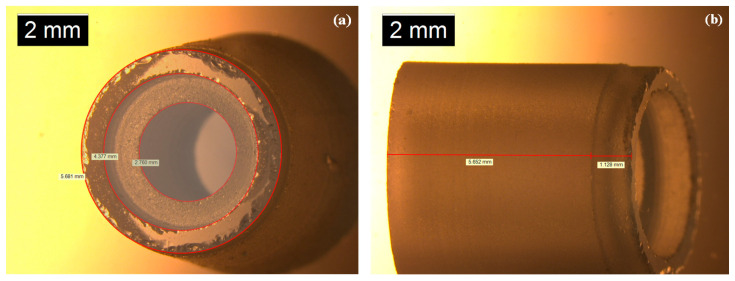
(**a**) Top view of a quartz tube. (**b**) Side view of a quartz tube.

Then, we used a quartz tube to support an unsupported GO film in order to make the sensing probe, and its top view and side view are shown in Figure 6.

**Figure 7:**
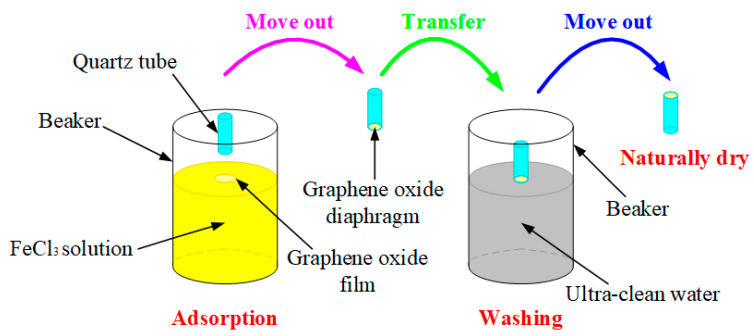
Operation of GO sensing diaphragm on a quartz tube.

It can be seen from Figure 6a that the inner diameter of the relatively larger circular hole of a quartz tube is approximately 4.377 mm. Then, we used the end face to adsorb an unsupported GO film from FeCl_3_ solution and immersed the GO diaphragm in ultra-clean water to remove residual FeCl_3_ solution, as shown in Figure 7. Firstly, we put the relatively larger circular hole of a quartz tube down, and then we placed this quartz tube vertically above a prepared GO film floating on FeCl_3_ solution surface. Next, we slowly moved the quartz tube down until it came into contact with the prepared GO film, and then we continued to move the quartz tube down for a short distance so that the prepared GO film was pasted on the quartz tube’s end face due to the effect of Van der Waals forces [16,17]. We then slowly lifted the quartz tube up and moved it away from FeCl_3_ solution surface. Then, we immersed the GO diaphragm in ultra-clean water to remove residual FeCl_3_ solution. At this time, the thickness and effective vibration diameter of the GO sensing diaphragm on this quartz tube were approximately 500 nm and 4.377 mm, respectively.

In order to match this relatively smaller circular hole of the quartz tube, we inserted an SMF with a standard ceramic ferrule with a diameter of 2.5 mm into this quartz tube through a high-resolution translation stage, thereby making this sensing probe. In fact, static operating point position can affect sensing probe sensitivity, and F-P cavity length can affect static operating point position [15,16,17,18,19,20,21,22,23]. Therefore, we needed to calculate and select the F-P cavity length of this sensing probe.

Because the end face reflectance R_1_ of the SMF and the inner surface reflectance R_2_ of the GO sensing diaphragm are less than 50%, expression (1) can be simplified as expression (3).

(3) $$R\left(\lambda ,l\right)=\frac{I_{R}\left(\lambda ,l\right)}{I_{0}}=2\sqrt{R_{1}R_{2}}\left(1-\cos\left(\frac{{4\pi n}_{0}l}{\lambda }\right)\right)$$

Therefore,

(4) $$I_{R}\left(\lambda ,l\right){=I}_{0}\cdot 2\sqrt{R_{1}R_{2}}\left(1-\cos\left(\frac{{4\pi n}_{0}l}{\lambda }\right)\right)$$

In fact, the sensing probe sensitivity, S, can be defined as the rate of interference light power I_R_(*λ*,*l*) to F-P cavity length *l* [20,23], namely

(5) $$S=\frac{{\mathrm{dI}}_{R}\left(\lambda ,l\right)}{dl}$$

Therefore, if the sensing probe sensitivity is maximized, then

(6) $$\frac{d^{2}I\left(\lambda ,l\right)}{dl^{2}}=0$$

At this time, output voltage signals of the proposed sensor cannot be distorted.

Therefore, according to expression (6), we can obtain F-P cavity length *l* as shown in expression (7).

(7) $$l=\lambda \left(\frac{2k+1}{8}\right)\;\left(k=1,2,3,\cdots \right)$$

It can be seen from expression (7) that there are countless F-P cavity lengths meeting the static operating point at optimal operating point position when the incident light wavelength is determined. However, for this sensing probe, working environment temperature can cause a change in F-P cavity length, which can make the static working point drift. Therefore, it is necessary to adjust the incident light wavelength to stabilize the static operating point at optimal operating point position by adjusting the incident light wavelength. In fact, too small of an F-P cavity length can make the free spectral range (FSR) of the proposed sensor too large [28], which can result in the inability to stabilize the static operating point at optimal operating point position. Therefore, the tuning range of the incident light wavelength should be greater than or equal to the FSR of this proposed sensor.

Therefore,

(8) $$\lambda_{2}{-\lambda }_{1}\geq K\cdot \mathrm{FSR}$$

and

(9) $$\mathrm{FSR}=\frac{\lambda_{0}^{2}}{2l}$$

Then,

(10) $$l\geq \frac{{K\lambda }_{0}^{2}}{2\left(\lambda_{2}{-\lambda }_{1}\right)}$$

In expressions (8)–(10), λ_1_ and λ_2_ represent the minimum value and the maximum value of the incident light wavelength, respectively; λ_0_ represents the average value of the incident light wavelength; K represents a redundancy factor, generally taken as 1.5.

In this experiment, the wavelength of the tunable laser was adjustable in the range of 1525 to 1565 nm; thus, according to expression (6), we can obtain the F-P cavity length range, namely, l≥44.815 μm.

In addition, because the longer the F-P cavity length, the more serious the transmission loss of the light incident into F-P cavity [16,17,18,19,20,21,22], additionally, the adjustment accuracy of high resolution translation stage is 0.5 μm. Thus, from the two perspectives, we have chosen *L* = 45.0 μm as the F-P cavity length.

At this time, we can obtain inequality (11) through expression (7)

(11) $$1525\;\mathrm{nm}\leq \frac{8l}{2k+1}\leq 1565\;\mathrm{nm}$$

When *L* = 45.0 μm, the range of k is 114.516 to 117.533. Because *k* is a positive integer, k can take 114, 115, 116 and 117, and when *k* = 116, the incident light wavelength is approximately 1545.06 nm; at this time, the adjustable range of the light wavelength output by the tunable laser can be applied to the maximum extent. Therefore, the incident light wavelength has been chosen as 1545.06 nm.

**Figure 8:**
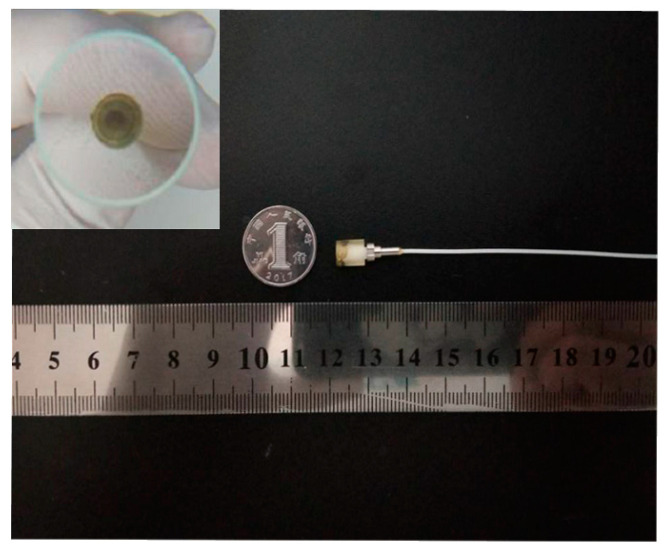
Actual size and shape of this sensing probe.

Therefore, we inserted an SMF with a standard ceramic ferrule into a suitable position inside this quartz tube through the high-resolution translation stage and glued this quartz tube and this SMF with UV glue, thereby obtaining this sensing probe as shown in Figure 8.

## 3. Results and Discussion

### 3.1. Performance Tests of the Proposed Sensor

**Figure 9:**
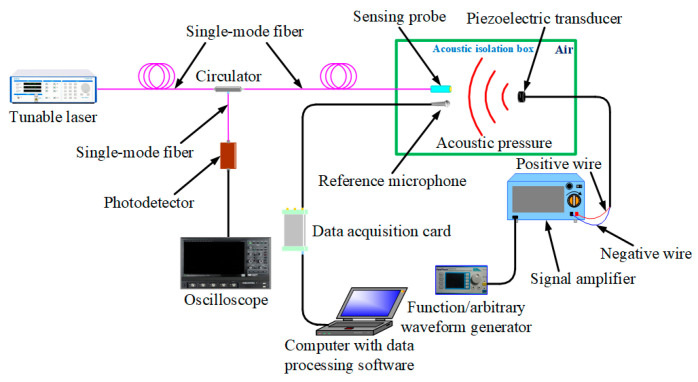
Platform for testing the proposed sensor performances.

Figure 9 shows the platform for testing this proposed sensor performances, which consists of this proposed sensor and an excitation system composed of a piezoelectric transducer, a signal amplifier and a waveform generator. In Figure 9, a microphone (MPA401) whose sensitivity is 5.0 mV/Pa as the reference microphone is placed side by side with the sensing probe to detect acoustic signals, and this sensing probe, the reference microphone and the piezoelectric transducer can be placed in an acoustic isolation box to eliminate the influence of environmental noise. Acoustic signals generated by this excitation system can act on this sensing probe, thereby causing this proposed sensor to work.

**Figure 10:**
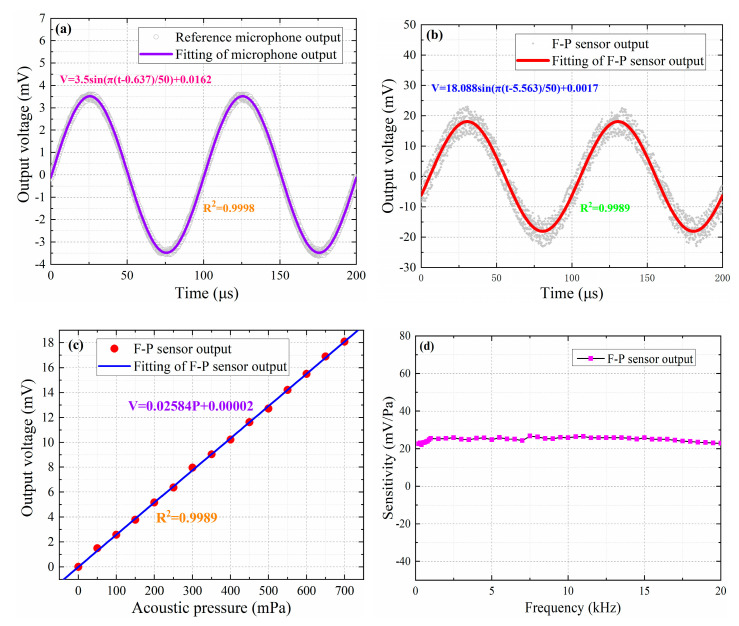
(**a**) Voltage signal output by the reference microphone at 10 kHz. (**b**) Voltage signal output by the proposed sensor at 10 kHz. (**c**) Acoustic-pressure response of the proposed sensor at 10 kHz. (**d**) Frequency response of the proposed sensor in the range of 200 Hz to 20 kHz.

Figure 10a,b show the output voltage signals of the reference microphone and this proposed sensor, respectively, when the frequency of sinusoidal acoustic signal generated by an excitation system is 10 kHz, and at this time, the acoustic-pressure size can be calculated by the reference microphone sensitivity and output voltage signal amplitude. Therefore, we used this method to obtain voltage amplitude values corresponding to 15 different acoustic-pressure values at 10 kHz, as shown in Figure 10c. It can be seen from Figure 10c that output voltage value linearly increases as acoustic pressure continues to increase, which indicates this proposed sensor has a greatly linear response to acoustic signals of different levels. Additionally, the proposed sensor sensitivity can be expressed as the ratio of output voltage signal amplitude to acoustic-pressure size [28]. Therefore, we used this platform to obtain the proposed sensor sensitivity at different frequencies, ranging from 200 Hz to 20 kHz, as shown in Figure 10d. It can be seen from Figure 10d that frequency response values have very small fluctuations in the range of 200 Hz to 20 kHz, which indicates that there is a flat frequency response in the range of 200 Hz to 20 kHz for this proposed sensor.

**Figure 11:**
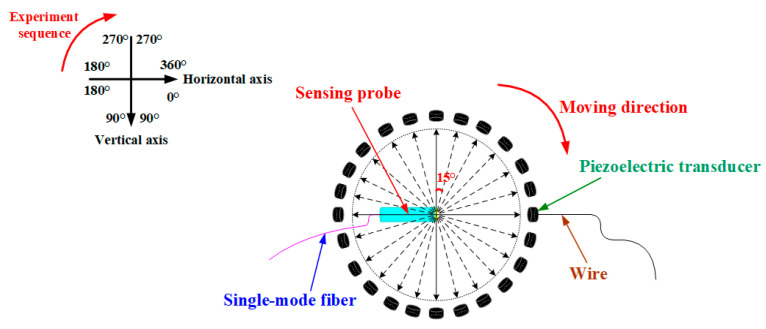
Directional test of this proposed sensor.

**Figure 12:**
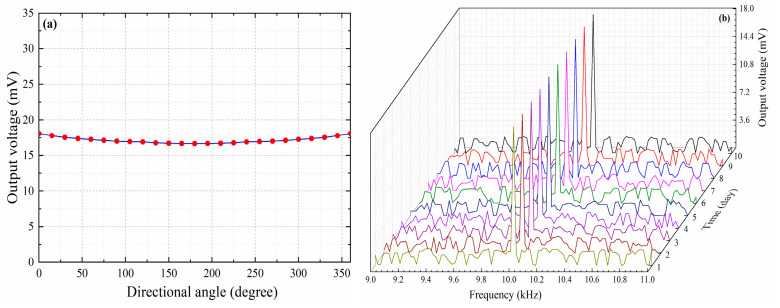
(**a**) Directional response of this proposed sensor. (**b**) Work stability of this proposed sensor during a ten-day test period.

Figure 11 shows the directional test of this proposed sensor. A sinusoidal acoustic signal with a frequency of 10 kHz was tested by this sensing probe every 15° clockwise, and the experimental results are shown in Figure 12a. It can be seen from Figure 12a that this proposed sensor has an approximately equal intensity to acoustic signals in different directions, which indicates that this proposed sensor is an omnidirectional sensor. Besides, we also used this proposed sensor to detect a sinusoidal acoustic signal with a frequency of 10 kHz for 10 consecutive days, and the frequency response values are shown in Figure 12b. It can be seen from Figure 12b that this proposed sensor has basically the same frequency response, which indicates that the proposed sensor has high working stability during a ten-day test period.

### 3.2. Experiment on the Detection of Partial Discharges in Air

**Figure 13:**
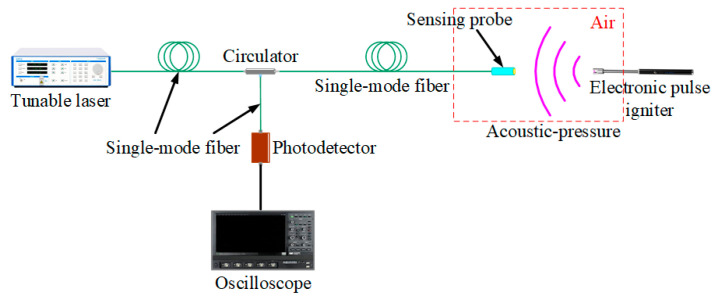
Particle discharge (PD) detection platform.

Figure 13 shows the PD detection platform. In this platform, PDs in air can be generated by an electronic pulse igniter [3,4,5,6,7]. Acoustic signals generated by PDs can act on this sensing probe, thereby causing this proposed sensor to work. Besides, we have kept the positions of the sensing probe and the electronic pulse igniter unchanged, and 20 repeated tests were implemented in order to obtain the representative size of the smallest PDs detected by this proposed sensor in air.

**Figure 14:**
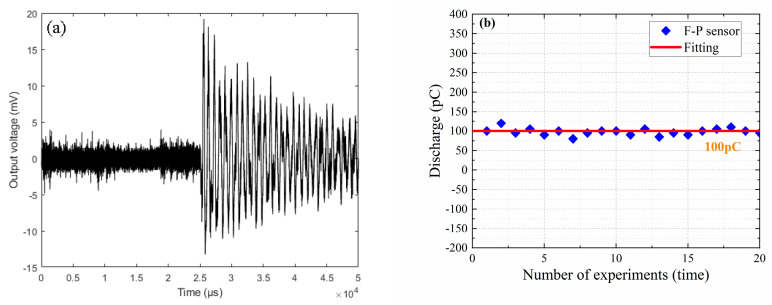
(**a**) PDs detected by this proposed sensor in air. (**b**) Minimum PD sizes corresponding to 20 experiments.

Figure 14a shows PDs detected by this proposed sensor in air. In Figure 14a, the noise voltage of this whole sensing system is shown to be approximately 1.7 mV, and this proposed sensor has a response voltage of 19.6 mV. Additionally, the size of PDs released by the electronic pulse igniter, according to its manufacturer, is approximately 1200 pC. Therefore, the minimum PD size detected by this proposed sensor in air was approximately 100 pC. Figure 14b shows the minimum PD sizes corresponding to 20 experiments. It can be seen from Figure 14b that the smallest PDs detected by this proposed sensor in air can, indeed, be considered as approximately 100 pC in size, which indicates that this proposed sensor has a relatively higher sensitivity for PD detection in air compared to other fiber-optic sensors [1,2,3,4,5,6,7].

## 4. Conclusions

In conclusion, an extrinsic fiber-optic F-P acoustic sensor based on a large-area and nanoscale GO diaphragm has been fabricated by an uncomplicated method. Performance tests indicated that this proposed sensor maintains a linear acoustic-pressure response and a flat frequency response in the range of 200 Hz to 20 kHz, while being an omnidirectional sensor and having high working stability during a ten-day test period. The PD detection result indicates that minimum PD size detected by this proposed sensor in air is approximately 100 pC, which demonstrates that this proposed sensor can greatly achieve high-sensitivity PD detection. Therefore, this proposed sensor has excellent potential for practical usage in high-sensitivity PD detection inside some power equipment.
